# Reliability of Large Language Model-Based Artificial Intelligence in AIS Assessment: Lenke Classification and Fusion-Level Suggestion

**DOI:** 10.3390/diagnostics15243219

**Published:** 2025-12-16

**Authors:** Cemil Aktan, Akın Koşar, Melih Ünal, Murat Korkmaz, Özcan Kaya, Turgut Akgül, Ferhat Güler

**Affiliations:** 1 Department of Orthopedics and Traumatology, Antalya Training and Research Hospital, Antalya 07100, Turkey; 2 Department of Orthopedics and Traumatology, Faculty of Medicine, Istanbul University, Istanbul 34093, Turkey; 3 Department of Orthopedics and Traumatology, İstinye University Medical Park TEM Hospital, Istanbul 34250, Turkey

**Keywords:** artificial intelligence, multimodal large language models, adolescent idiopathic scoliosis, Lenke classification, deep learning

## Abstract

**Background:** Accurate deformity classification and fusion-level planning are essential in adolescent idiopathic scoliosis (AIS) surgery and are traditionally guided by Cobb angle measurement and the Lenke system. Multimodal large language models (LLMs) (e.g., ChatGPT-4.0; Claude 3.7 Sonnet, Gemini 2.5 Pro, DeepSeek-R1-0528 Chat) are increasingly used for image interpretation despite limited validation for radiographic decision-making. This study evaluated the agreement and reproducibility of contemporary multimodal LLMs for AIS assessment compared with expert spine surgeons. **Methods:** This single-center retrospective study included 125 AIS patients (94 females, 31 males; mean age 14.8 ± 1.9 years) who underwent posterior instrumentation (2020–2024). Two experienced spine surgeons independently performed Lenke classification (including lumbar and sagittal modifiers) and selected fusion levels (UIV–LIV) on standing AP, lateral, and side-bending radiographs; discrepancies were resolved by consensus to establish the reference standard. The same radiographs were analyzed by four paid multimodal LLMs using standardized zero-shot prompts. Because LLMs showed inconsistent end-vertebra selection, LLM-derived Cobb angles lacked a common anatomical reference frame and were excluded from quantitative analysis. Agreement with expert consensus and test–retest reproducibility (repeat analyses one week apart) were assessed using Cohen’s κ. Evaluation times were recorded. **Results:** Surgeon agreement was high for Lenke classification (92.0%, κ = 0.913) and fusion-level selection (88.8%, κ = 0.879). All LLMs demonstrated chance-level test–retest reproducibility and very low agreement with expert consensus (Lenke: 1.6–10.2%, κ = 0.001–0.036; fusion: 0.8–12.0%, κ = 0.003–0.053). Claude produced missing outputs in 17 Lenke and 29 fusion-level cases. Although LLMs completed assessments far faster than surgeons (seconds vs. ~11–12 min), speed did not translate into clinically acceptable reliability. **Conclusions:** Current general-purpose multimodal LLMs do not provide reliable Lenke classification or fusion-level planning in AIS. Their poor agreement with expert surgeons and marked internal inconsistency indicate that LLM-generated interpretations should not be used for surgical decision-making or patient self-assessment without task-specific validation.

## 1. Introduction

Adolescent idiopathic scoliosis (AIS) is a three-dimensional spinal deformity of unknown etiology, defined by a coronal Cobb angle greater than 10° and typically manifesting between 10 and 18 years of age [1,2]. Affecting 2–3% of adolescents worldwide, AIS shows clear sex-related patterns, with a substantially higher risk of progression in females—particularly when curves exceed 30° [3,4,5,6]. Curvatures ≥50° at skeletal maturity tend to progress and may cause pain, postural imbalance, cosmetic concerns, and functional limitations [7,8]. Accordingly, early recognition and accurate radiographic evaluation remain essential for predicting progression and optimizing treatment decisions.

Cobb angle remains the fundamental radiographic metric for quantifying coronal curve magnitude in AIS and is measured between the most tilted superior and inferior end vertebrae of the structural curve [9]. The Lenke classification system provides a standardized three-component framework—curve type, lumbar modifier, and sagittal thoracic modifier—for describing deformity patterns and determining appropriate surgical strategies [10]. Fusion-level planning, typically expressed as the selection of the upper and lower instrumented vertebrae (UIV and LIV), is critical for achieving postoperative coronal and sagittal balance and for minimizing complications such as distal adding-on or junctional failure [11,12]. These foundational concepts form the basis of deformity assessment and guide all subsequent decisions regarding operative management in AIS.

In spine surgery, classification systems such as the Lenke system are critical for determining fusion levels and preserving motion segments. However, both Cobb angle measurement and Lenke typing show considerable interobserver variability, even among experienced surgeons, largely due to subjective identification of endplates [9,13]. Manual Cobb measurement is not only prone to 4–8° variation but is also time-consuming, often requiring up to 15–20 min per case. These inconsistencies may alter Lenke classification and subsequently influence fusion-level planning, prompting increasing interest in automated or computer-assisted tools to enhance reproducibility and reduce evaluation time.

Parallel to these developments, the widespread use of generative artificial intelligence (AI) systems has reshaped how clinicians and patients access medical information. Multimodal large language models (LLMs)—such as ChatGPT, Claude, Gemini, and DeepSeek—are now capable of processing both text and images, and are increasingly consulted for conditions like scoliosis. Yet, emerging orthopedic evidence suggests that while these systems provide reasonably accurate general explanations, their performance declines in specialized clinical tasks, including radiographic interpretation, surgical planning, and deformity classification [14,15,16,17,18,19]. Moreover, reflecting a Dunning–Kruger-type cognitive pattern, LLMs may produce overly confident but incorrect answers [20]. Although deep learning-based convolutional neural networks have achieved promising results in automated scoliosis measurement [18], multimodal LLMs have not been systematically evaluated for Cobb angle quantification, Lenke typing, or fusion-level recommendation. This represents a significant knowledge gap given their increasing availability and unregulated clinical use.

Therefore, the present study aims to objectively evaluate the reliability of contemporary multimodal LLMs in interpreting scoliosis radiographs. Specifically, we assessed their performance in Cobb angle measurement, Lenke classification, and fusion-level prediction, comparing these outputs with expert surgeon assessments and evaluating consistency across repeated LLM queries. We hypothesized that despite generating fluent and persuasive responses, LLM performance would remain inferior to expert accuracy and reliability, underscoring the need for cautious interpretation when these systems are used in clinical decision-making or accessed directly by patients.

## 2. Materials and Methods

### 2.1. Study Design and Ethical Approval

This single-center retrospective observational study was conducted at X Hospital using anonymized preoperative conventional spinal radiographs of patients diagnosed with adolescent idiopathic scoliosis (AIS) who subsequently underwent posterior spinal instrumentation between 2020 and 2024. The study protocol complied with the principles of the Declaration of Helsinki and received approval from the Institutional Ethics Committee of Antalya Training and Research Hospital Scientific Ethical Committee for Medical Research (Approval No: 8/7; 8 May 2025). Because only de-identified preoperative imaging data were used, and no intervention was performed, the requirement for written informed consent was waived in accordance with institutional policy.

### 2.2. Patient Selection

All eligible patients were identified through a systematic search of the institutional PACS archive. The two research investigators screened all consecutive patients aged 10–18 years with a confirmed diagnosis of adolescent idiopathic scoliosis (AIS) who subsequently underwent posterior corrective fusion between January 2020 and December 2024. Only preoperative radiographs were evaluated for this study.

Inclusion criteria were as follows:

(1) Availability of complete and good-quality preoperative standing anteroposterior (AP), lateral, and right/left side-bending radiographs;
(2) Planned treatment with posterior spinal instrumentation.

Exclusion criteria included congenital vertebral anomalies, neuromuscular scoliosis, age <10 or >18 years, non-surgical management (e.g., bracing), and incomplete radiographic datasets.

Radiographs not meeting quality requirements were excluded but not replaced, ensuring that the final dataset represented a consecutive, non-selected cohort and minimizing selection bias. All eligible radiographs were retrieved from the PACS archive, anonymized, and independently verified by two researchers (Table 1).

### 2.3. Radiographic Protocol

All radiographs included in this study were originally acquired as part of the routine institutional scoliosis imaging protocol and not specifically for research purposes. Full-length standing anteroposterior (AP) and lateral spine radiographs were obtained with patients in the upright position, knees fully extended, and in neutral frontal alignment using a flat-panel digital radiography system (D-EVO Suite II 180, Fujifilm Corporation, Tokyo, Japan) at a standard source-to-detector distance of 2 m. For lateral projections, patients placed their hands on their clavicles to minimize humeral overlap and standardize sagittal balance evaluation. Right and left side-bending radiographs were routinely obtained preoperatively in surgical candidates to assess curve flexibility.

All radiographs were exported in lossless PNG format with a native matrix size of approximately 3000 × 1500 pixels and a spatial resolution of 0.15–0.20 mm/pixel. Images were stored as 12-bit grayscale files, preserving the full dynamic range of the original Fujifilm D-EVO Suite II radiography system. These image properties were kept identical for all datasets and for all uploads to the multimodal LLMs to ensure consistent and reproducible visual input across models.

**Figure 1:**
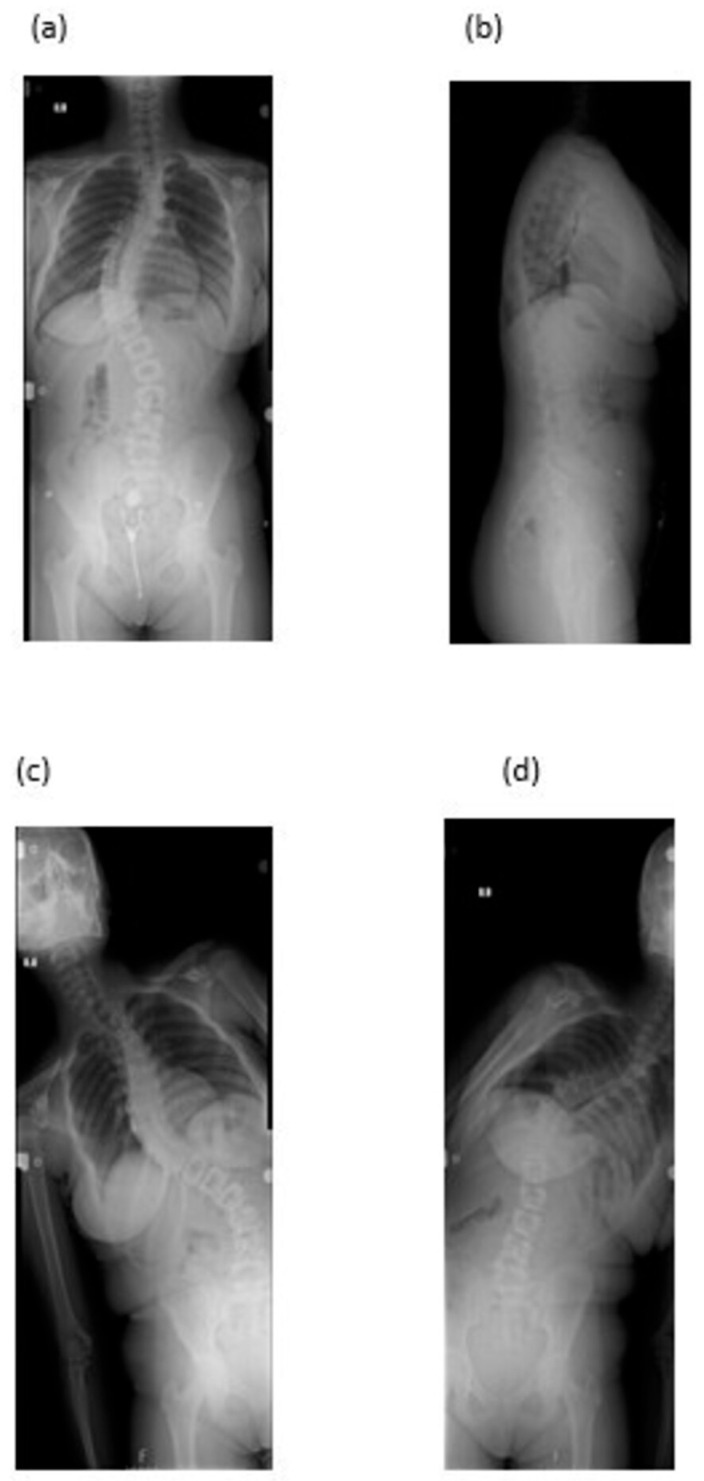
Representative full-length spine radiographs uploaded to the AI systems for analysis: (**a**) Standing anteroposterior (AP) view; (**b**) standing lateral view; (**c**) right bending; and (**d**) left bending radiographs obtained according to the standardized institutional scoliosis imaging protocol. Images were acquired using a flat-panel digital radiography system (D-EVO Suite II 180, Fujifilm Corporation, Tokyo, Japan) at a 2 m source-to-detector distance and exported in PNG format for AI evaluation.

For the present analysis, these previously acquired images were retrospectively retrieved in PNG format from the PACS archive. Only lossless-format images were included to ensure full preservation of anatomical detail. Radiographs demonstrating rotation artifacts, positioning errors, incomplete visualization, or inadequate resolution were excluded. Final image eligibility was independently verified by two senior spine surgeons (Figure 1).

Although all radiographs were uploaded to the LLMs at their full native resolution, it should be noted that some multimodal AI interfaces may internally downsample images upon upload. No manual cropping, resizing, or compression was performed by the investigators at any stage of data handling. Standard PACS-generated left/right (L/R) orientation markers were present on standing AP radiographs; however, manual review confirmed that none of the models relied on these textual markers or exhibited text-driven hallucination. All observed errors originated from geometric misinterpretation of spinal anatomy rather than from the presence of L/R labels or other non-anatomical cues.

### 2.4. Radiographic Measurements by Spine Surgeons

For each eligible case, two experienced spine surgeons independently measured the Cobb angles using digital drawing tools within the PACS system. Lenke classification was performed according to established criteria, with both the lumbar modifier and sagittal modifier included to ensure complete curve characterization. Each surgeon also proposed the appropriate fusion level—defining the upper and lower instrumented vertebrae—based on Lenke recommendations and standard surgical planning principles.

To address sagittal plane evaluation in greater detail, thoracic kyphosis was measured between the superior endplate of T5 and the inferior endplate of T12 on standing lateral radiographs. Based on Lenke criteria, sagittal thoracic modifiers were assigned as follows: “−” for hypokyphosis (<10°), “N” for normal kyphosis (10–40°), and “+” for hyperkyphosis (>40°). Both surgeons independently determined these values prior to consensus review.

Similarly, lumbar modifiers (A, B, and C) were assigned on standing AP radiographs using the relationship between the center sacral vertical line (CSVL) and the apical lumbar vertebra. Modifier A was defined when the CSVL passed between the pedicles of the apical lumbar vertebra, modifier B when it touched one pedicle, and modifier C when it completely fell outside the pedicle margins.

Interobserver reliability for Lenke type and fusion level determination was quantified using Cohen’s κ coefficient. A consensus Lenke classification was subsequently established through joint review, whereas individual fusion level proposals were retained for agreement analysis. This consensus served as the ground truth for all AI model comparisons. This consensus Lenke classification served as the ground truth for all subsequent AI comparisons.

### 2.5. Fusion Level Selection Criteria

Fusion level determination was performed independently by two experienced spine surgeons according to standard deformity correction principles endorsed by the Scoliosis Research Society (SRS). Selection of the upper instrumented vertebra (UIV) was based on identifying the proximal structural curve and extending instrumentation to the neutral and stable vertebra to minimize postoperative shoulder imbalance and proximal junctional issues.

Determination of the lower instrumented vertebra (LIV) incorporated multiple established criteria, including: (1) the stable vertebra on standing AP radiographs, (2) the last touched vertebra by the center sacral vertical line (CSVL), (3) the lower-end vertebra of the structural thoracic or thoracolumbar/lumbar curve, and (4) flexibility on side-bending radiographs. Particular emphasis was placed on avoiding distal adding-on by selecting an LIV that aligns with the stable and neutral vertebra whenever possible.

Because fusion level selection in AIS surgery remains an area of active debate within the SRS—especially regarding optimal LIV determination in Lenke 1 and 2 curves—final fusion decisions were individualized and based on surgeon judgment rather than a rigid algorithm. Disagreements between the two surgeons were resolved through consensus review, and these consensus fusion levels were used as the reference standard for AI model comparison.

### 2.6. Artificial Intelligence Evaluation

All radiographs used for AI analysis were derived from the exact preoperative images reviewed by the surgeons. To ensure identical visual input, the same PACS views used by the surgeons were captured as high-resolution screenshots and exported in lossless PNG format. This procedure guaranteed that all AI models evaluated precisely the same content measured by the surgeons.

Each patient’s AP, lateral, and right/left bending radiographs were subsequently uploaded as anonymized PNG files to four generative artificial intelligence systems—ChatGPT (OpenAI), Claude (Anthropic), DeepSeek, and Gemini (Google). To maintain consistency, all four models received identical instructions and uniform task phrasing. Analyses were conducted using the paid (subscription-based) versions of each system to guarantee access to their most capable multimodal reasoning models. All analyses were performed using the paid multimodal versions of each system available on August 10, 2025. The specific model builds used were ChatGPT (GPT-4.o, version 2025-08-10), Claude 3.7 Sonnet (version 2025-08-10), Gemini 2.5 Pro (version 2025-08-10), and DeepSeek-R1-0528 Chat (version 2025-08-10).

A standardized three-step instruction set was applied as follows:

1. Measure Cobb angles and report each curve with its vertebral limits, angle in degrees, and structurality (structural vs. non-structural).

Example: PT: T2–T6: 33° (S); MT: T6–T12: 59° (S); L: L1–L4: 39° (NS).

2. Perform Lenke classification, providing curve type, lumbar modifier, and sagittal thoracic modifier as a single three-character code.

Example: 2BN.

3. Recommend appropriate fusion levels according to Lenke guidelines.

Example: T2–L2.

AI models were provided with the same lateral radiographs and explicitly instructed to determine the sagittal modifier as part of the Lenke classification code, ensuring methodological consistency with surgeon assessments.

To assess test–retest reliability, each AI model analyzed the entire dataset twice, with a one-week interval between sessions. All outputs were exported, archived, and standardized for subsequent statistical evaluation. Missing or incomplete AI outputs—defined as cases where the model failed to produce Cobb angles, Lenke subtype, or fusion-level predictions—were recorded and excluded from κ computations, but were reported descriptively to quantify output reliability.

### 2.7. Prompt Design and Interaction Method

To ensure full reproducibility, prompt design was standardized across all LLMs. A single zero-shot prompt was used for all analyses. No prompt optimization, few-shot prompting, chain-of-thought exemplars, or iterative refinement was applied in order to reflect real-world clinical usage and avoid artificially enhancing model performance.

All models received the same three-step instruction set described above. The ideal expected output format consisted of the following:

(1) Cobb angles with explicit upper- and lower-end vertebrae and structurality;
(2) A three-character Lenke code (curve type, lumbar modifier, sagittal modifier);
(3) A recommended fusion range (UIV–LIV) consistent with Lenke guidelines.

All interactions were performed through the official paid web interfaces of each AI system rather than via API access. Representative correct, incorrect, and incomplete model outputs are provided in Supplementary File S1.

All prompts were written and delivered in English, and all model outputs were generated in English.

### 2.8. Assessment of Cobb Angle Feasibility in LLM Outputs

Because Cobb angle measurement requires fixed identification of the superior and inferior end vertebrae, we first evaluated whether each LLM produced anatomically valid and internally consistent end-vertebra selections. During preliminary data extraction, all four models demonstrated substantial variability in landmark identification, often selecting different end vertebrae for the same radiograph and occasionally choosing levels far outside the structural curve. Owing to this instability, Cobb angles generated by the LLMs could not be aligned to a common anatomical reference frame and were therefore deemed unsuitable for statistical comparison. For this reason, Cobb angle outputs were recorded descriptively but excluded from all quantitative analyses, while detailed failure rates were reported separately in Section 4.

### 2.9. Handling of Incomplete or Missing LLM Outputs

To ensure transparent evaluation of model performance, all instances in which an LLM produced incomplete or missing outputs were systematically documented. For Claude, these failures fell into two distinct categories: (1) explicit safety refusals, in which the model stated that it could not provide diagnostic interpretation of medical images, and (2) technical interruptions, where a response was initiated but remained truncated or incomplete without a refusal message. Each affected case was reviewed and categorized accordingly. This distinction allowed us to differentiate safety-driven restrictions from genuine processing or reasoning failures during multimodal image interpretation.

### 2.10. Measurement Time Evaluation

To evaluate procedural efficiency, the duration required for each radiographic assessment was quantified for both human raters and AI systems. Two experienced spine surgeons performed Cobb angle measurement, Lenke classification (including lumbar and sagittal modifiers), and fusion-level determination using the digital measurement tools of the PACS interface. For every radiograph, the time from image display to completion of all three tasks was recorded in seconds using an integrated screen-timer application. The measurement times of the two surgeons were averaged to obtain a representative clinician benchmark. AI models were evaluated under identical timing conditions; the duration from image upload to final output generation was recorded in seconds. For AI systems, the interval from image upload to completion of the model’s final output was recorded. Each AI model performed the full three-step analysis twice, one week apart, and the mean of the two runs was used for comparative time analysis, while run-to-run variability was quantified separately to assess temporal stability.

## 3. Statistical Analysis

All statistical analyses were performed using IBM SPSS Statistics version 29.0 (IBM Corp., Armonk, NY, USA). Descriptive statistics were used to summarize demographic characteristics, Lenke subtype distribution, and fusion-level ranges.

Interobserver agreement between the two spine surgeons, test–retest reliability within each artificial intelligence (AI) model, and agreement between AI-generated outputs and the surgeons’ consensus reference standard were evaluated using Cohen’s κ coefficient, with 95% confidence intervals (CIs) calculated for all κ estimates. Percent agreement values were additionally reported to provide descriptive context for κ results, given the known sensitivity of κ to class prevalence and marginal distributions. Agreement strength was interpreted according to the Landis and Koch scale.

Exact matching was required for all agreement analyses. Any discrepancy in Lenke curve type, lumbar modifier (A/B/C), sagittal modifier (−/N/+), or fusion-level selection was considered categorically incorrect rather than partially accurate, as even minor deviations in these parameters may directly influence surgical planning and treatment decisions in adolescent idiopathic scoliosis.

Pairwise comparisons between AI systems were prespecified and performed using z-tests for independent κ coefficients, based on standard asymptotic variance estimates, to determine whether any model demonstrated superior agreement in Lenke classification or fusion-level determination. Missing or incomplete AI outputs were excluded from κ calculations but were quantified and reported separately as an indicator of model output reliability.

Because Cobb angle measurement is entirely dependent on consistent identification of superior and inferior end vertebrae, and because preliminary analysis demonstrated substantial inter- and intra-model variability in end-vertebra selection, LLM-derived Cobb angles were deemed anatomically inconsistent and unsuitable for quantitative comparison. Accordingly, Cobb angle outputs were excluded from all statistical analyses, and landmark identification failure rates were reported descriptively.

To address the potential influence of class imbalance on κ values, additional agreement metrics were calculated. Balanced accuracy was computed for Lenke classification to account for unequal subtype prevalence. Gwet’s AC1 statistic was also calculated as an alternative chance-corrected agreement measure less sensitive to prevalence effects, enabling verification that low κ values reflected true disagreement rather than a statistical artifact.

Evaluation time (seconds) for surgeons and AI systems was treated as a continuous variable and summarized using means, standard deviations, medians, and interquartile ranges (IQR). Given the non-normal distribution and extreme magnitude differences between human and AI assessment times, between-group differences were quantified using Cliff’s delta (δ) effect size, interpreted according to established thresholds. Time distributions were visualized using boxplots and violin plots, with logarithmic scaling applied to accommodate differences exceeding two orders of magnitude.

All statistical tests were two-tailed, and a *p*-value < 0.05 was considered statistically significant.

## 4. Results

A total of 125 patients (94 females and 31 males; mean age, 14.8 ± 1.9 years; range, 10–18 years) diagnosed with adolescent idiopathic scoliosis (AIS) and subsequently treated with posterior instrumentation were included in this study. All demographic information corresponded to the preoperative cohort from which the radiographs were obtained.

Cobb angles of the proximal thoracic (PT), main thoracic (MT), and thoracolumbar/lumbar (TL/L) curves demonstrated substantial variability across patients and curve patterns. However, because AI models frequently selected inconsistent upper- and lower-end vertebrae, direct numeric comparisons were invalid; therefore, Cobb angles were excluded from statistical analysis. Subsequent evaluations, therefore, focused exclusively on Lenke classification and fusion level determination, which were consistently defined by both spine surgeons and the AI models. All 125 cases proceeded to agreement analysis.

Two experienced spine surgeons independently evaluated all 125 AIS cases according to standardized criteria. For Lenke classification, the interobserver percent agreement was 92.0% with a Cohen’s κ value of 0.913, indicating almost perfect reliability based on the Landis and Koch interpretation scale. Similarly, for fusion level determination, the agreement rate was 88.8% with κ = 0.879, representing substantial consistency between observers. When both surgeons assigned identical Lenke classifications and fusion levels, these results were directly accepted. In cases with discrepant ratings, a consensus decision was reached through joint review. All final classifications, whether directly concordant or resolved through consensus, served as the reference standard for AI comparison.

**Figure 2:**
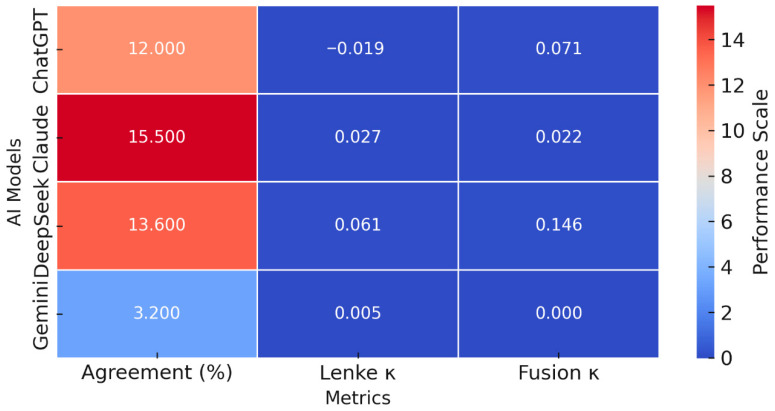
Heatmap of agreement and reliability metrics across AI models. Heatmap illustrating agreement rates (%) and Cohen’s κ values for Lenke classification and fusion level determination across the four AI models. Darker red tones represent relatively higher agreement or reliability, whereas blue tones indicate low or near-zero performance. Although DeepSeek demonstrated slightly higher reproducibility than the other systems (Lenke κ = 0.061; fusion κ = 0.146), these values remained far below clinically acceptable thresholds. Gemini exhibited the lowest overall consistency, with minimal agreement (3.2%) and κ values approaching zero for both metrics, highlighting the limited ability of current multimodal LLMs to generate stable or reliable scoliosis classifications.

Each AI model reanalyzed the same set of anonymized radiographic images one week later using identical standardized prompts to assess temporal stability and test–retest reliability. Despite identical radiographs and identical instructions, test–retest κ values for all AI systems clustered near zero, indicating chance-level reproducibility. Temporal inconsistency was particularly pronounced in the assignment of Lenke modifiers, both lumbar (A/B/C) and sagittal (−/N/+), and in the selection of upper and lower instrumented vertebrae for fusion planning (Figure 2). These findings demonstrate that all AI models exhibited poor temporal reproducibility despite identical input conditions.

### 4.1. Failure of End-Vertebra Identification and Its Impact on Cobb Angle Measurement

Before analyzing Cobb angle outputs, we evaluated whether each LLM correctly identified the superior and inferior end vertebrae. Across all 125 AIS cases (250 vertebra assignments), the models exhibited substantial landmark selection errors. Incorrect end-vertebra identification occurred in 73.6–80.8% of all assignments across the four LLMs. More importantly, 30.4–33.6% of all vertebrae selected by the models were classified as grossly incorrect, defined as choices ≥3 vertebral levels away from the expert-defined reference. Error rates for each model are summarized in Table 2.

Because Cobb angle magnitude is entirely dependent on accurate and consistent end-vertebra identification, these landmark-selection failures rendered all LLM-derived Cobb angles structurally invalid and unsuitable for statistical analysis.

**Figure 3:**
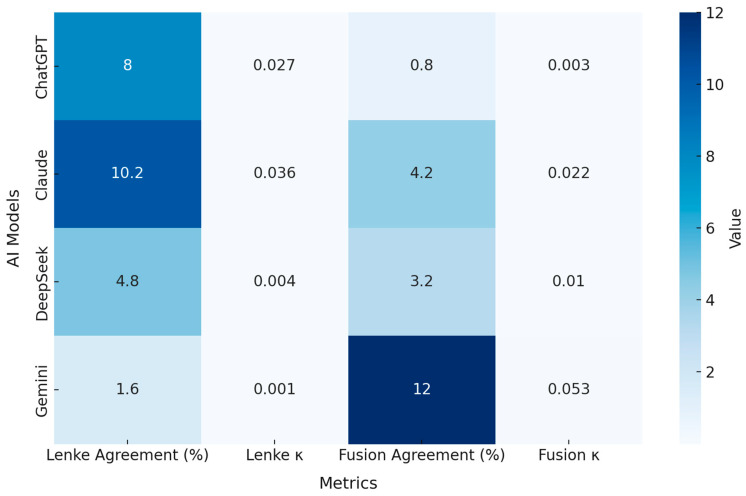
Heatmap illustrating agreement rates (%) and Cohen’s κ values for Lenke classification and fusion level determination across the four AI systems. Darker tones represent relatively higher agreement or reliability, while lighter tones indicate near-zero performance. Despite minor numerical differences among models, all systems showed minimal agreement with surgeon consensus, with κ values ranging from 0.001 to 0.053. Gemini demonstrated the highest fusion-level agreement (12.0%), but its κ value (0.053) remained far below clinically acceptable reliability thresholds.

When the second-time AI outputs were compared with the surgeons’ consensus, all models demonstrated very low concordance for both Lenke classification and fusion level determination (Table 3). Lenke classification agreement ranged from only 1.6% to 10.2%, with corresponding κ values between 0.001 and 0.036, indicating virtually no agreement beyond chance. Fusion level recommendations showed similar inaccuracy, with agreement rates between 0.8% and 12.0% and κ values from 0.003 to 0.053. Although Gemini yielded the numerically highest κ for fusion level recommendations (κ = 0.053), this value remained well below levels indicative of reliable classification performance (Figure 3). Notably, Claude generated missing or incomplete outputs in 17 Lenke cases and 29 fusion-level assignments, further reducing its overall reliability. Overall, none of the AI systems approached expert-level accuracy on any metric.

Claude’s missing outputs were primarily due to explicit safety refusals, in which the model stated that it could not provide diagnostic interpretation of medical images. In a smaller number of cases, the model initiated a response but failed to complete the output, resulting in truncated text. No system crashes occurred.

### 4.2. Subtype-Specific Performance

To further characterize model behavior, accuracy was analyzed for each individual Lenke subtype (Supplementary Table S1). Subtype-level accuracy remained extremely low across all models, with most subgroups demonstrating 0% correct classification. Even in the most frequently encountered categories (such as 1AN and 1BN), accuracy ranged only from 0% to 42.9%, and no model achieved reliable performance in any subtype. Error patterns were systematic rather than random, with recurrent misidentification of structural curves, lumbar modifiers, and sagittal modifiers. These findings indicate that the poor overall accuracy observed in the primary analysis persisted uniformly across all clinically relevant Lenke categories and was not driven by a small number of outlier cases.

Pairwise statistical comparisons of κ values were conducted to assess whether any AI model outperformed the others in Lenke classification or fusion level determination (Table 4). No statistically significant differences were detected for any model pair across either metric (all *p*-values 0.69–0.98). Although minor numerical variations were observed—such as Gemini’s slightly higher κ for fusion (0.053)—these differences did not represent meaningful improvements in agreement performance. Overall, κ values for all models remained clustered near zero, confirming that none of the AI systems demonstrated superior agreement relative to one another or to expert consensus.

### 4.3. Additional Agreement Metrics to Address Class Imbalance

Because κ values can be affected by heterogeneous class distributions, additional agreement metrics were calculated to validate model performance. The prevalence of Lenke types in the study cohort is summarized in Table 5. Balanced accuracy remained low across all models (0.168–0.204), indicating performance close to chance even after correcting for class imbalance. Gwet’s AC1 values were similarly negative (−1.63 to −3.12), confirming that low agreement was not attributable to κ deflation but instead reflected true disagreement with expert consensus. Confusion matrices for each model, illustrating the distribution of misclassification patterns across Lenke types, are provided in Supplementary Figures S1–S4.

### 4.4. Qualitative Analysis of Confident Failures

Despite clear radiographic features and structured prompting, the multimodal LLM occasionally produced Lenke classifications that directly contradicted established criteria while maintaining a confident explanatory tone.

**Figure 4:**
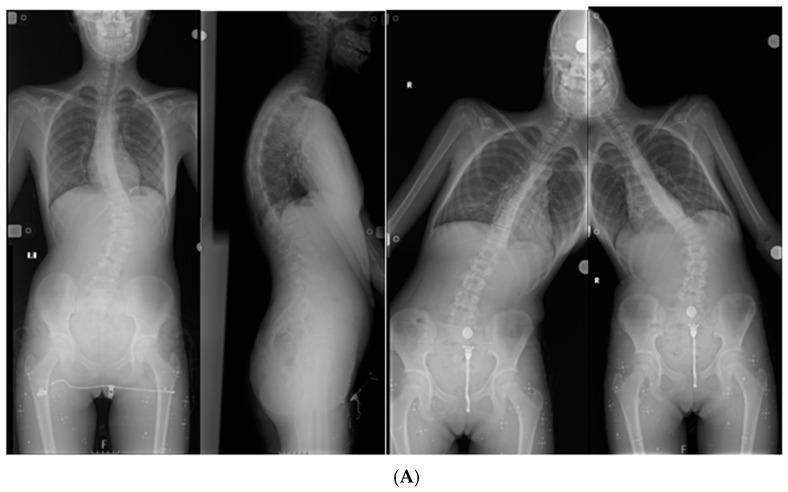

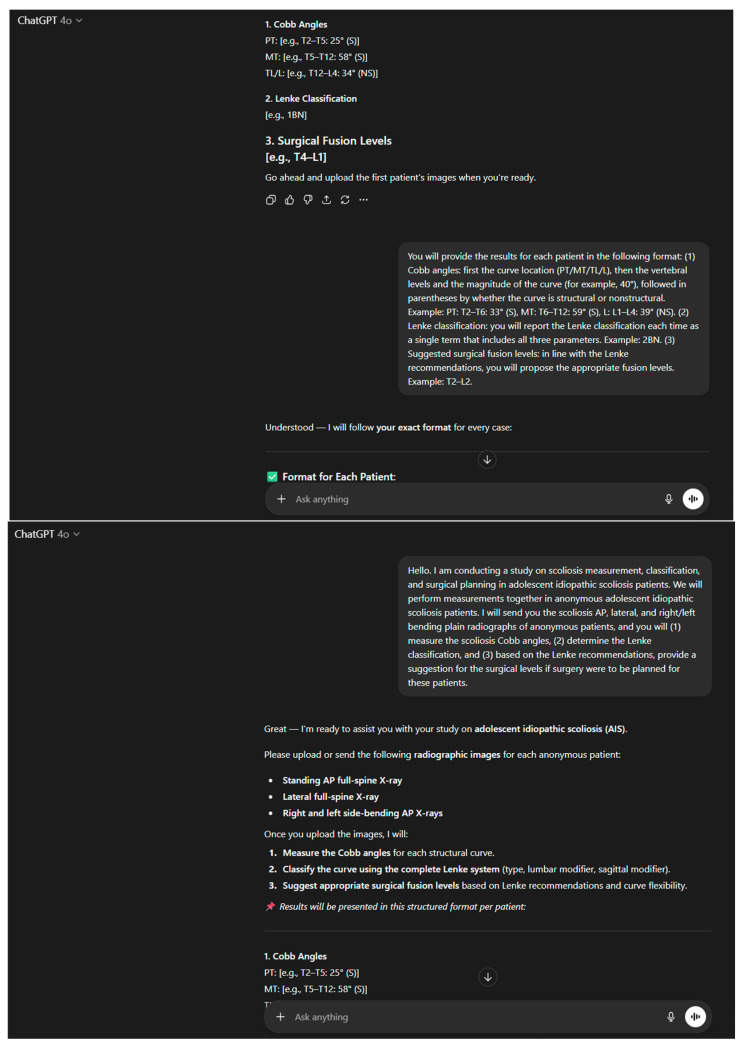

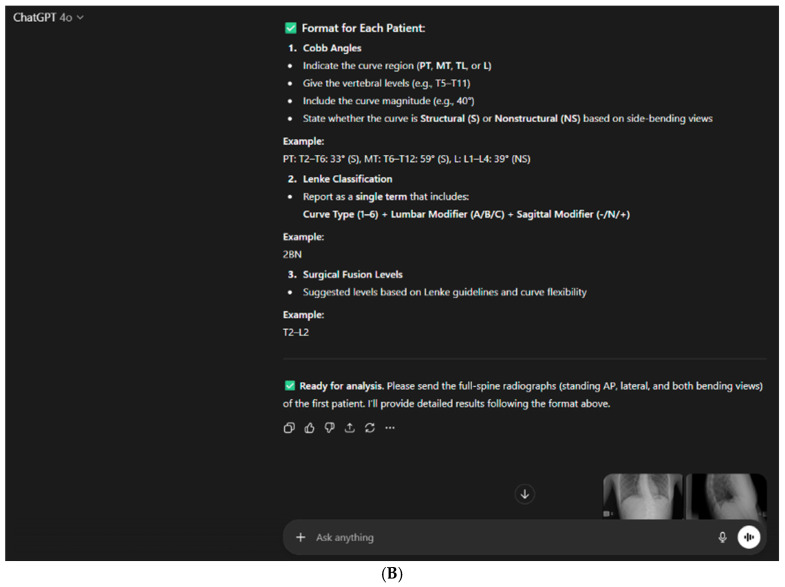

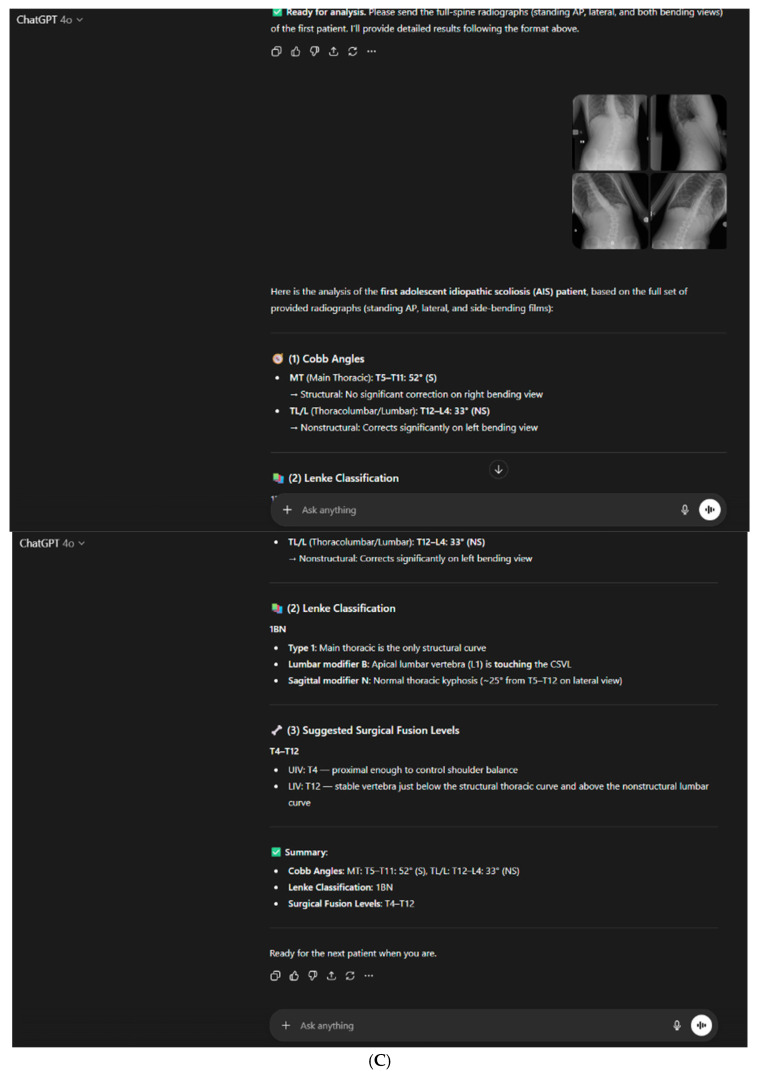
Example of a confident failure (“hallucination”) by the multimodal LLM: (**A**) Standing posteroanterior radiograph of an adolescent idiopathic scoliosis case unanimously classified as Lenke type 5 by two orthopedic surgeons. (**B**) Prompt provided to the multimodal LLM requesting Lenke classification. (**C**) Model-generated response confidently classifying the curve as Lenke type 1, accompanied by anatomically inconsistent reasoning.

Figure 4 illustrates a representative case that was unanimously classified as Lenke type 5 by both orthopedic surgeons, yet was classified by the LLM as Lenke type 1. The model’s explanation incorrectly interpreted the lumbar curve as non-structural and failed to recognize the dominant thoracolumbar/lumbar curve pattern, which are defining characteristics of Lenke type 5 curves.

This misclassification represents a confident failure, as the output was delivered with high certainty despite violating fundamental principles of Lenke classification. Such hallucination-like behavior provides qualitative insight into the low inter-method agreement observed in the quantitative analysis.

### 4.5. Time-to-Completion (AI)

**Figure 5:**
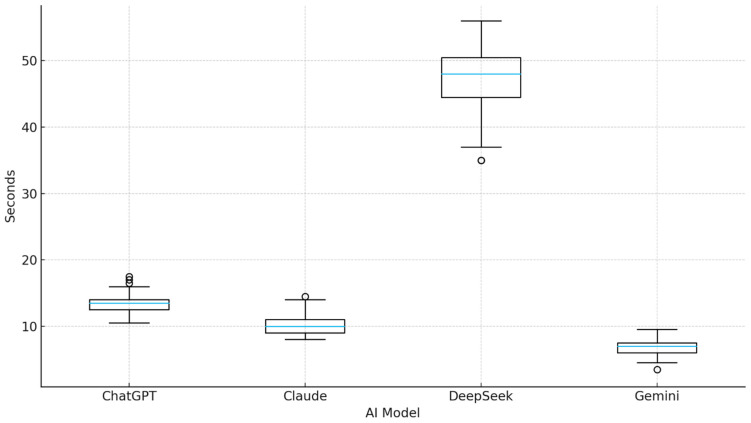
Time-to-completion distribution across AI models. Boxplots display the distribution of processing times for four AI systems. The horizontal line within each box indicates the median value, while box boundaries represent the interquartile range (IQR). Whiskers denote the range of non-outlier observations, and individual points mark statistical outliers. Gemini exhibited the shortest and most consistent completion times, followed by Claude and ChatGPT, which maintained similarly narrow IQRs. In contrast, DeepSeek demonstrated markedly prolonged and highly variable processing durations, with extended whiskers and multiple outliers, indicating reduced temporal stability relative to other models.

Time-to-completion profiles differed markedly across the four AI systems, as illustrated in Figure 5. Gemini demonstrated the fastest and most consistent performance, with a median processing time of 7.0 s (IQR: 6.0–7.5). ChatGPT also performed rapidly, yielding a median of 13.5 s (IQR: 12.5–14.0), while Claude showed slightly slower but still moderate processing times (median 10.0 s, IQR: 9.0–11.0). In contrast, DeepSeek exhibited substantially prolonged and highly variable completion times, with a median of 48.0 s (IQR: 44.5–50.5), far exceeding the other models. The markedly wider IQR and dispersion in DeepSeek processing times indicated not only slower performance but also reduced temporal stability. Conversely, Gemini and ChatGPT demonstrated narrower IQRs and fewer outliers, reflecting more consistent runtime behavior.

### 4.6. Surgeon Assessment Time

The evaluation times recorded by the two spine surgeons showed a high degree of consistency, with per-case durations ranging from 600 to 890 s. Surgeon 1 exhibited a mean completion time of 731.36 ± 112.55 s (median: 720 s, IQR: 620–820 s), while Surgeon 2 demonstrated a comparable mean time of 695.68 ± 86.80 s (median: 680 s, IQR: 620–720 s). When averaged on a per-case basis, the combined surgeon assessment time was 713.52 ± 74.26 s (median: 710 s, IQR: 650–765 s), indicating that expert manual completion of Cobb measurements, Lenke classification (including modifiers), and fusion-level selection typically requires approximately 11–12 min.

**Figure 6:**
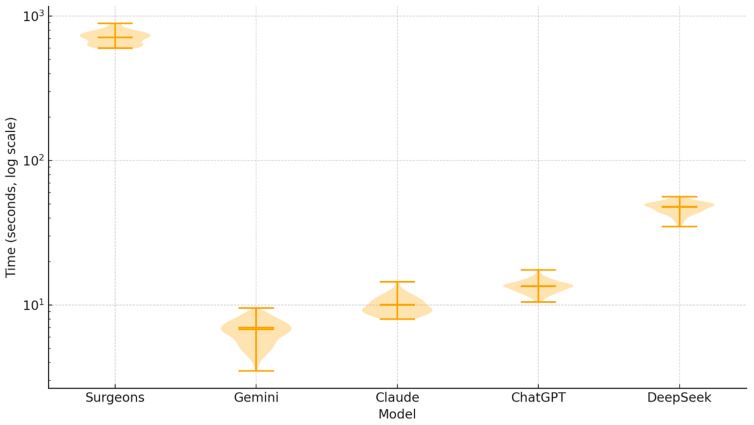
Violin plot illustrating the distribution of evaluation times for surgeons and AI systems on a logarithmic scale. Surgeons demonstrated a narrow yet substantially elevated time distribution (≈600–890 s), reflecting consistent but time-intensive manual assessment. In contrast, Gemini, Claude, and ChatGPT exhibited compact distributions with extremely rapid completion times (<15 s). DeepSeek showed a broader and higher distribution with greater variability, though still maintaining over a 15-fold speed advantage compared with surgeons. Median values are indicated by central horizontal lines, mean values by thicker bands, and shaded contours represent kernel density estimates. Logarithmic scaling was applied to demonstrate differences exceeding two orders of magnitude across models.

When contrasted with these surgeon-derived durations, all AI systems completed the same tasks in exceptionally shorter periods. Gemini was the fastest model (6.76 ± 1.22 s), followed by Claude (10.03 ± 1.42 s) and ChatGPT (13.49 ± 1.30 s), while even the slowest model, DeepSeek, still completed evaluations in 47.54 ± 4.42 s—more than 15 times faster than human evaluators. Effect sizes were extreme (Cliff’s delta ≈±1 across all surgeon–AI comparisons), indicating near-maximal differences in processing time favoring AI systems (Figure 6).

## 5. Discussion

In this study, we conducted a comprehensive evaluation of contemporary multimodal large language models in the radiographic assessment of adolescent idiopathic scoliosis. Although all AI systems were tested using identical high-resolution images and standardized prompts, none of the models demonstrated clinically meaningful agreement with expert surgeons for Lenke classification or fusion-level planning. Concordance rates rarely exceeded 10%, and κ values consistently clustered near zero, indicating performance no better than chance. Test–retest reliability was likewise poor, with all LLMs producing markedly inconsistent classifications and fusion recommendations across repeated analyses, in stark contrast to the almost perfect interobserver reliability observed between spine surgeons. While AI models completed evaluations within seconds—substantially faster than the 11–12 min required for expert assessment—this computational speed did not translate into accuracy, reproducibility, or clinically interpretable decision-making. These findings highlight a critical mismatch between the rapid output generation of current multimodal LLMs and their inability to deliver reliable deformity classification or surgical planning information in AIS. Despite their substantial speed advantage, this rapid processing offered no clinical benefit because the outputs lacked reliability, reproducibility, and anatomical accuracy. In AIS management, where surgical planning relies on precise curve characterization and carefully justified fusion levels, consistency is far more critical than speed. Therefore, the current generation of multimodal LLMs cannot be considered suitable for clinical decision-making despite their efficiency, as their rapid analyses are fundamentally undermined by high rates of classification error and internal variability.

In the present study, Cobb angle values generated by LLM-based systems were excluded from statistical analysis because the models selected different superior and inferior end vertebrae across repeated measurements. Since Cobb angle quantification is entirely dependent on fixed end-vertebra identification, variability in these anatomical landmarks renders the resulting angles structurally non-comparable. This inconsistency prevented alignment of measurements to a shared anatomical reference framework, producing a non-homogeneous dataset unsuitable for valid statistical analysis. This phenomenon is consistent with prior evidence showing that end-vertebra selection is a major source of measurement variability even among experienced clinicians [21]. The fundamental limitation underlying these inconsistencies is that current multimodal LLMs lack reliable geometric landmark recognition and therefore cannot consistently identify vertebral boundaries. This deficiency led directly to unstable end-vertebra selection and, consequently, invalid Cobb angle estimations.

Recent advances in artificial intelligence and machine learning have led to a rapid expansion of their applications in orthopedics, with promising results reported in domains such as osteoarthritis grading, tumor classification, and fracture detection [22,23]. These technologies have demonstrated the potential to enhance diagnostic accuracy and streamline clinical workflows. In adolescent idiopathic scoliosis (AIS), emerging studies similarly suggest that AI may support screening, facilitate earlier diagnosis, aid in predicting curve progression, and enable personalized rehabilitation strategies. However, research specifically focused on AI applications for AIS remains in an early developmental stage, and most related studies have been published within the past five years, reflecting a rapidly evolving yet still exploratory field [24].

Despite this growing interest, a major barrier to the adoption of AI tools in orthopedic practice is the limited understanding of how different computational approaches operate. Terms such as “artificial intelligence,” “machine learning,” “deep learning,” and, more recently, “large language models (LLMs)” are often used interchangeably in the literature, contributing to conceptual confusion. In reality, AI represents the broadest umbrella, while machine learning (ML) refers to algorithms that learn from large datasets. Deep learning (DL), a subset of ML, relies on artificial neural networks (ANNs), with convolutional neural networks (CNNs) serving as the dominant architecture for image feature extraction and computer vision tasks. LLMs, by contrast, constitute a specialized subclass of DL models trained on massive language corpora; although originally designed for text-based reasoning, modern multimodal LLMs incorporate image-processing components that enable visual interpretation. Understanding these distinctions is essential when evaluating model performance, as general-purpose LLMs differ fundamentally from task-specific vision models traditionally used in radiographic analysis.

This conceptual ambiguity, combined with the growing accessibility of large language models (LLMs), has led patients and clinicians to use these systems for image interpretation far more frequently than anticipated. Yet, the reliability of models originally designed for text-based reasoning when applied to real clinical radiographs remains largely unknown. In conditions such as scoliosis—where the tolerance for diagnostic error is minimal—this uncertainty represents a significant clinical risk and underscores the need for systematic evaluation of multimodal LLMs’ visual reasoning capabilities. Motivated by this need, our study demonstrates that current LLMs fail to produce consistent, reproducible, and surgically meaningful outputs in the assessment of AIS.

The reliability of the Lenke classification represents a critical foundation for interpreting our findings, as it serves as the principal framework guiding surgical decision-making in AIS. Originally introduced by Lenke in 2001 to define curve types and determine the extent of fusion [10], this system has generally demonstrated high inter- and intra-observer reliability across multiple studies [11,12,25], although moderate consistency has also been reported in certain series [26]. In our cohort, two experienced spine surgeons achieved a 92.0% agreement rate and a κ value of 0.913 for Lenke typing, indicating “almost perfect” reliability. Fusion-level determination showed similarly high concordance (agreement 88.8%, κ = 0.879). These results highlight the robustness of the Lenke classification when applied by trained specialists using standardized radiographs and reinforce its role as the most dependable clinical reference for AIS surgical planning. Importantly, establishing such a high-fidelity human reference allowed for a direct and meaningful comparison with LLM-based outputs, underscoring the considerable performance gap observed between expert surgeons and current multimodal LLMs.

Previous work has shown that the interobserver reliability of the Lenke classification is not uniform across studies, institutions, or observer groups. Ogon et al. reported moderate interobserver agreement (κ ≈ 0.62) and substantial intraobserver reliability (κ ≈ 0.73) in a multisurgeon cohort, with most disagreements arising from evaluation of upper thoracic structurality and lumbar modifiers [11]. Richards et al. similarly demonstrated fair-to-good interobserver reliability (κ ≈ 0.50–0.64) when the full three-component Lenke scheme was applied to non-premeasured radiographs, noting improved consistency when only curve type was classified [26]. Niemeyer et al. found that both Lenke and King classifications show poor-to-fair interobserver agreement on raw radiographs, but improve to good-to-excellent reliability once Cobb angles are pre-marked and observers are highly trained [27]. In contrast, Hosseinpour-Feizi et al. reported high agreement levels in a single-center cohort evaluated by expert readers [25].

Taken together, these studies demonstrate that Lenke reliability is context-dependent and influenced by measurement workflow, radiograph preparation, and observer experience—rather than uniformly excellent. This variability provides important context for the almost perfect agreement observed between the two expert surgeons in our study.

Accurate selection of fusion levels is essential in adolescent idiopathic scoliosis (AIS) surgery because both over-fusion and under-fusion carry clinically significant risks. Overly extensive fusion sacrifices mobile segments and may increase biomechanical loading on adjacent levels, potentially accelerating adjacent segment degeneration and reducing postoperative spinal mobility. On the other hand, inappropriate selection of the lower instrumented vertebra (LIV) has been strongly associated with postoperative deformity progression, particularly the distal adding-on phenomenon and coronal decompensation, which can worsen alignment and may necessitate revision surgery. Improper LIV selection—defined as stopping fusion proximal to key stable landmarks such as the last substantially touched vertebra (LSTV) or stable vertebra (SV)—has been shown to increase the risk of distal adding-on and junctional complications in AIS patients. Studies demonstrate that fusing to levels proximal to these landmarks is correlated with higher distal adding-on rates, whereas selecting an LIV that encompasses established radiographic criteria (such as CSVL alignment and stable vertebral alignment) reduces this risk and improves radiographic outcomes. These findings underscore the importance of precise preoperative determination of fusion levels, guided by established radiographic parameters and evidence-based selection criteria, to minimize complications and optimize surgical outcomes in AIS [28,29].

A final methodological consideration concerns the prompting strategy used in this study. We intentionally employed a standardized zero-shot three-step prompt to simulate how an average clinician or end-user might interact with a multimodal LLM in routine practice. However, growing evidence indicates that more sophisticated prompting approaches—particularly Chain-of-Thought (CoT) prompting—can substantially enhance reasoning performance in large language models by explicitly guiding intermediate analytical steps [30]. Providing exemplar Lenke classifications or curated step-by-step reasoning chains might therefore have improved the models’ ability to identify curve types and fusion levels. Importantly, no alternative prompts were piloted in this study, and our findings reflect LLM performance under unsupervised, real-world usage conditions rather than under optimized prompt-engineering scenarios. Future work may explore whether CoT-based prompting or few-shot examples meaningfully alter LLM behavior in spinal deformity classification.

An additional factor influencing model performance is the sensitivity of multimodal LLMs to prompt design. Because these systems rely on text-conditioned reasoning rather than explicit visual–geometric computation, even subtle variations in wording, description style, or instruction specificity can lead to markedly different interpretations of the same radiograph. To minimize this variability, our study used fully standardized prompts and identical high-resolution images for all models; however, this did not eliminate inconsistent outputs, as several LLMs still produced divergent Lenke classifications and fusion recommendations across repeated trials. This prompt-dependent instability has been described as an inherent limitation of LLM inference and further underscores why current multimodal language–vision models cannot yet be considered reliable decision-support tools in AIS assessment.

An additional limitation was the incomplete or missing responses produced by certain models, most notably Claude. This phenomenon likely reflects the model’s difficulty in extracting clinically relevant radiographic features when image resolution, curvature complexity, or vertebral landmarks fall below its visual-confidence threshold. In such situations, Claude’s conservative inference strategy—combined with safety filters that restrict detailed surgical recommendations—results in output truncation. Consequently, even when identical radiographs and prompts were used, some models failed to deliver complete Lenke classifications or fusion-level plans, further highlighting their current clinical unreliability.

Traditional deep learning systems for scoliosis analysis have been developed on structured radiographic datasets and are primarily used by orthopedic specialists. For example, Xie et al. demonstrated that task-specific computer vision models can accurately measure Cobb angles and sagittal parameters when imaging conditions and anatomical cues are strictly standardized [31]. However, these systems operate under controlled environments and cannot reflect the way clinical decisions are made in everyday practice. In contrast, large language models (LLMs) such as ChatGPT, Claude, DeepSeek, and Gemini are publicly accessible tools increasingly consulted by patients and non-specialist clinicians, yet their ability to perform complex classification tasks has remained largely untested. In this context, our study provides the first systematic evaluation of LLM performance in Lenke classification and fusion planning. Notably, despite their widespread availability, the LLMs we tested demonstrated substantially lower reliability compared with experienced spinal surgeons, with considerable variability, especially in identifying structural curves and determining appropriate fusion levels. These findings underscore the gap between specialized deep learning systems and general-purpose LLMs and highlight the need for careful consideration when these models are used for clinical guidance.

Previous studies have demonstrated that deep learning models trained directly on scoliosis radiographs can achieve high accuracy in automated measurements using techniques such as multi-stage ensemble networks, keypoint detection, and vertebral segmentation [10,32,33,34]. However, these systems fundamentally rely on geometric feature extraction from controlled imaging environments—an ability that large language models inherently lack. Because LLMs operate through verbal reasoning rather than spatial computation, they are not equipped to represent the three-dimensional complexity of scoliotic deformities. Moreover, scoliosis classification in clinical practice extends beyond numerical measurements; surgeons integrate structural curve characteristics, curve stiffness, compensatory patterns, and sagittal alignment into a holistic decision-making framework [10]. The inability of LLMs to approximate this nuanced clinical judgment likely explains the limited reliability and marked variability observed in our study, particularly in identifying structural curves and determining fusion levels. The inconsistent outputs generated when identical radiographs were re-evaluated further highlight the ambiguity-prone and non-reproducible nature of LLM decision-making, consistent with prior analyses on LLM self-consistency under uncertainty [35]. Despite increasing interest in AI for scoliosis, the overall volume and methodological quality of current literature remain limited, underscoring the need for systematic and bibliometric evaluations to identify research gaps and guide future methodological development.

One of the most recent advances in scoliosis-related AI is the development of deep learning models capable of estimating curve severity from non-radiographic images, such as raster stereographic back surfaces [36] or even single smartphone photographs [32]. These studies highlight the impressive performance of task-specific convolutional neural networks while also emphasizing their dependence on controlled imaging workflows, curated training datasets, and specialized computational infrastructure. In contrast, multimodal large language models such as GPT-4o, Claude 3.7 Sonnet, Gemini 2.5 Pro, and DeepSeek-R1-0528 Chat are not designed for radiological measurement yet are increasingly used by both clinicians and patients to interpret spinal radiographs. Unlike CNN-based scoliosis solutions, LLMs lack calibrated measurement modules, anatomical landmark detectors, and Lenke-specific decision pathways. Our findings further demonstrate that the visual-geometric reasoning capabilities of current multimodal LLMs remain markedly insufficient for scoliosis-specific tasks, performing far below the accuracy, consistency, and structural recognition required for clinical use. These limitations resulted in poor agreement with expert assessments and substantial internal variability, indicating that the current generation of LLMs cannot yet support clinically meaningful AIS classification or surgical planning.

A critical implication of our findings is the potential risk posed when patients or non-specialist clinicians rely on LLM-generated interpretations for self-assessment. Multimodal LLMs are increasingly used to analyze personally uploaded radiographs, yet our results demonstrate that these models frequently produce inaccurate, inconsistent, or incomplete classifications—even when provided with standardized, high-quality imaging. Such errors may lead patients to misjudge the severity of their deformity, misunderstand the need for surgery, or develop unnecessary anxiety or false reassurance. Given the narrow safety margin in AIS decision-making, unsupervised use of AI for diagnostic interpretation represents a substantial clinical risk and underscores the importance of keeping radiographic evaluation within the domain of trained specialists. Unlike engineering contexts where distance-based error metrics may be informative, scoliosis classification and fusion-level planning require exact agreement because even small deviations alter clinical decision-making. Therefore, partial accuracy metrics are not appropriate or clinically meaningful for the Lenke system or surgical planning tasks.

Taken together, our findings highlight that general-purpose multimodal LLMs currently fall short of the performance levels reported in prior studies using task-specific deep learning models for scoliosis assessment. The present LLM versions exhibit inadequate visual-geometric reasoning, poor agreement with expert assessments, and significant internal inconsistency even when evaluating the same radiographs—limitations that pose risks not only in clinical decision-making but also when patients use these tools for self-interpretation. Although multimodal models continue to evolve rapidly, safe integration into clinical workflows requires rigorous, task-specific, and prospective validation. Future efforts should prioritize hybrid frameworks that combine the geometric precision of computer vision with the contextual reasoning of language models, supported by large, scoliosis-specific datasets. Until such advances are achieved, LLM-generated outputs should be interpreted with caution by both clinicians and patients and must not replace expert clinical judgment.

This study has a number of significant strengths. Firstly, it presents one of the most comprehensive evaluations directly comparing the performance of multimodal large language models in interpreting AIS radiographs with that of expert spine surgeons, and examines their real-world use on radiographic images. Secondly, it evaluates model reliability within a multidimensional framework, focusing not only on accuracy metrics but also on both internal consistency tests and expert agreement analysis; this approach provides a more clinically meaningful assessment of performance. Thirdly, the evaluation of current LLM versions from different manufacturers and architectures offers a broad and up-to-date perspective on the current capabilities and limitations of these models.

### Sample Size Considerations

This study included 125 AIS cases from a single tertiary spine center, which is a modest cohort size compared with contemporary recommendations for formal development or validation of AI-based prediction models in healthcare. Recent methodological work has emphasized that robust training and validation of clinical prediction models often require sample sizes in the order of hundreds to thousands of patients, with explicit a priori justification [37]. However, our study did not aim to train, fine-tune, or validate a new prediction model; instead, it was designed as an exploratory benchmarking analysis of four off-the-shelf multimodal LLMs under realistic clinical imaging conditions. Within this context, a cohort of 125 consecutively treated surgical AIS patients is typical for single-center spine surgery research and was sufficient to demonstrate that agreement with expert consensus clustered near chance, with very low κ values and narrow confidence intervals. Nevertheless, our findings should not be interpreted as a definitive validation study, and larger, multicenter cohorts will be essential for any future formal validation of scoliosis-specific AI systems.

The single-center design of this study may limit the generalizability of our findings. Interpretation of the Lenke classification can vary across surgeons and institutions due to differences in training, experience, and institutional practice patterns. Although our two experienced spine surgeons demonstrated almost perfect agreement, this level of concordance may not be replicated in more heterogeneous or multicenter environments. Therefore, the reference standard generated in this study may reflect center-specific expertise, which should be considered when interpreting the comparative performance of LLM-based systems.

In addition to these center-related considerations, however, certain limitations must also be acknowledged. The study relied on static radiographic images rather than 3D reconstructions, which may affect the interpretability of complex deformities. Furthermore, although we examined several LLM variants, model outputs may change over time as developers update training protocols; thus, our findings reflect performance at a specific point in technological evolution. Our evaluation was conducted using the most recent paid versions of the included LLMs; however, as these models were tested in their unmodified, general-purpose forms, their performance may not reflect the capabilities of systems specifically trained for scoliosis-related tasks.

A further fundamental limitation of current multimodal LLMs is their inability to reliably identify anatomical landmarks or perform geometric reasoning based on radiographic structures. Unlike computer-vision models specifically trained on vertebral segmentation or keypoint detection tasks, LLMs do not extract spatial features from images in a structured manner. As a result, they struggle to localize pedicles, endplates, apices, or curve transition zones—elements that are essential for Lenke classification and fusion level planning. This lack of anatomically grounded spatial computation likely contributed to the inconsistent selection of end vertebrae, misidentification of structural curves, and unstable fusion recommendations observed in our study. These limitations reflect the current gap between general-purpose multimodal LLMs and specialized geometric or landmark-based vision systems used in deformity assessment.

## 6. Conclusions

In conclusion, widely used multimodal large language models currently do not provide clinically reliable outputs for AIS radiograph interpretation, including Lenke classification and fusion-level planning. Across repeated analyses with identical images and prompts, these systems demonstrated marked internal inconsistency and poor agreement with expert surgeon reference standards, and some models produced incomplete outputs. These findings indicate a substantive risk if LLM-generated interpretations are used for clinical decision-making or patient self-assessment in scoliosis care. While ongoing advances in AI may improve performance, safe clinical integration will require rigorous, task-specific validation and likely hybrid frameworks that combine explicit geometric measurement (computer vision) with language-based reasoning. Until such systems are prospectively validated, multimodal LLM outputs should be interpreted with caution and must not replace expert clinical judgment.

## Figures and Tables

**Table 1:** Characteristics of the scoliosis dataset used for LLM evaluation.

Variable	Value	
Total patients	**125**	
Age, mean ± SD (range)	**14.8 ± 1.9 (10–18)**	
Sex distribution	**94 females (75.2%), 31 males (24.8%)**	
Lenke classification distribution	1AN: 17 cases 1BN: 9 cases 1CN: 7 cases 2AN: 8 cases 2BN: 33 cases 2CN: 24 cases	3BN: 5 cases 4BN: 3cases 5BN: 3 cases 6CN: 16 cases
Radiograph types included	Standing AP, lateral, right bending, left bending	
Number of radiographic files	**4 images per patient → 500 total images**	
Consensus fusion levels	**T2–L3** (*n* = 39) **T2–L4** (*n* = 32) **T2–L2** (*n* = 27) **T3–L2** (*n* = 10)	**T3–L3** (*n* = 7) **T4–L1** (*n* = 4) **T2–L1** (*n* = 3) **T4–L2** (*n* = 3)

**Table 2:** End-vertebra identification error rates across AI models.

Model	Incorrect (%)	Grossly Incorrect (%)
Claude	77.2%	32.0%
Gemini	73.6%	30.4%
ChatGPT	80.4%	33.6%
DeepSeek	80.8%	33.6%

**Table 3:** Agreement between AI systems and doctor consensus for Lenke classification and fusion level determination.

AI System	Parameter	Total Cases	Complete Pairs (N)	Missing AI	% Agreement	Cohen’s κ
ChatGPT	LC	125	125	0	8.0	0.027
	Fusion level	125	125	0	0.8	0.003
Claude	LC	125	108	17	10.2	0.036
	Fusion level	125	96	29	4.2	0.022
DeepSeek	LC	125	125	0	4.8	0.004
	Fusion level	125	124	1	3.2	0.010
Gemini	LC	125	125	0	1.6	0.001
	Fusion level	125	125	0	12.0	0.053

Note: Percent agreement represents the proportion of identical classifications between AI systems and expert consensus. Cohen’s κ reflects agreement beyond chance. LC: Lenke classification. “Complete pairs” represent the number of radiographs for which both AI and surgeon consensus labels were available. Missing outputs were predominantly observed in Claude, which failed to generate a classification in 17 Lenke cases and 29 fusion-level cases. Percent agreement reflects the proportion of identical assignments, whereas Cohen’s κ quantifies agreement beyond chance.

**Table 4:** Pairwise comparisons of Cohen’s κ values between AI systems for Lenke classification and fusion level determination.

Comparison	Parameter	κ_1_	κ_2_	z	*p*-Value	Significant (*p* < 0.05)
ChatGPT vs. Claude	Lenke	0.027	0.036	−0.068	0.9456	No
	Fusion	0.003	0.022	−0.139	0.8892	No
ChatGPT vs. DeepSeek	Lenke	0.027	0.004	0.181	0.8563	No
	Fusion	0.003	0.010	−0.055	0.9561	No
ChatGPT vs. Gemini	Lenke	0.027	0.001	0.205	0.8378	No
	Fusion	0.003	0.053	−0.394	0.6936	No
Claude vs. DeepSeek	Lenke	0.036	0.004	0.243	0.8083	No
	Fusion	0.022	0.010	0.088	0.9300	No
Claude vs. Gemini	Lenke	0.036	0.001	0.265	0.7907	No
	Fusion	0.022	0.053	−0.228	0.8200	No
DeepSeek vs. Gemini	Lenke	0.004	0.001	0.024	0.9812	No
	Fusion	0.010	0.053	−0.338	0.7353	No

Pairwise z-tests were applied to evaluate whether any model demonstrated significantly higher agreement than the others. No statistically significant differences were detected across any model pair for either classification task (all *p*-values > 0.05).

**Table 5:** Prevalence of Lenke curve types in the study cohort.

Lenke Type	*n*	Prevalence (%)
1	48	38.4%
2	34	27.2%
3	12	9.6%
4	5	4.0%
5	22	17.6%
6	4	3.2%

## Data Availability

The data that support the findings of this study are available from the corresponding author upon reasonable request. The data are not publicly available due to institutional policy and privacy restrictions.

## Supplementary Materials

The following supporting information can be downloaded at: https://www.mdpi.com/article/10.3390/diagnostics15243219/s1, Figure S1: Confusion matrix demonstrating agreement between ChatGPT predictions and expert Lenke classifications. Off-diagonal clustering indicates widespread misclassification across curve types; Figure S2: Confusion matrix for Claude compared with expert Lenke classifications. The model shows inconsistent identification of curve types, with substantial errors across multiple categories; Figure S3: Confusion matrix illustrating DeepSeek’s Lenke classification performance. Misclassification is frequent and distributed across nearly all Lenke types, reflecting limited discriminatory accuracy; Figure S4: Confusion matrix for Gemini predictions relative to expert classification. The matrix shows biased prediction patterns and high misclassification rates, indicating poor alignment with ground truth; File S1: Representative correct, incorrect, and incomplete model outputs illustrating fusion-level recommendations generated according to the Lenke classification guidelines; Table S1: Subtype-Specific Accuracy of AI Models in Lenke Classification.

## Abbreviations

AIS	Adolescent idiopathic scoliosis
AI	Artificial intelligence
ANN	Artificial neural network
AP	Anteroposterior
CNN	Convolutional neural network
DL	Deep learning
LLM	Large language model
ML	Machine learning
MT	Main thoracic
PACS	Picture Archiving and Communication System
PNG	Portable Network Graphics
PT	Proximal thoracic
TL/L	Thoracolumbar/lumbar
UIV	Upper instrumented vertebra
LIV	Lower instrumented vertebra
κ (kappa)	Cohen’s kappa coefficient
IQR	Interquartile range
