# Supplementary material for: Reliability of Large Language Model-Based Artificial Intelligence in AIS Assessment: Lenke Classification and Fusion-Level Suggestion
Source: Diagnostics (Basel). 2025 Dec 16;15(24):3219. doi: 10.3390/diagnostics15243219 (PMC12731402; doi:10.3390/diagnostics15243219)
Supplement: Supplementary file 1 [file diagnostics-15-03219-s001.zip › Supplementary Fig. Legends S1-S2-S3-S4.pdf]

Supplementary Figure S1.

Confusion matrix demonstrating agreement between ChatGPT predictions and expert Lenke classifications. Off-diagonal clustering indicates widespread misclassification across curve types.

Supplementary Figure S2.

Confusion matrix for Claude compared with expert Lenke classifications. The model shows inconsistent identification of curve types, with substantial errors across multiple categories.

Supplementary Figure S3.

Confusion matrix illustrating DeepSeek's Lenke classification performance. Misclassification is frequent and distributed across nearly all Lenke types, reflecting limited discriminatory accuracy.

Supplementary Figure S4.

Confusion matrix for Gemini predictions relative to expert classification. The matrix shows biased prediction patterns and high misclassification rates, indicating poor alignment with ground truth.
