# Supplementary material for: Reliability of Large Language Model-Based Artificial Intelligence in AIS Assessment: Lenke Classification and Fusion-Level Suggestion
Source: Diagnostics (Basel). 2025 Dec 16;15(24):3219. doi: 10.3390/diagnostics15243219 (PMC12731402; doi:10.3390/diagnostics15243219)
Supplement: Supplementary file 1 [file diagnostics-15-03219-s001.zip › Supplementary File S1.pdf]

ChatGPT 4o

Hello. I am conducting a study on scoliosis measurement, classification, and surgical planning in adolescent idiopathic scoliosis patients. We will perform measurements together in anonymous adolescent idiopathic scoliosis patients. I will send you the scoliosis AP, lateral, and right/left bending plain radiographs of anonymous patients, and you will (1) measure the scoliosis Cobb angles, (2) determine the Lenke classification, and (3) based on the Lenke recommendations, provide a suggestion for the surgical levels if surgery were to be planned for these patients.

Perfect, I'm ready to assist you with your **adolescent idiopathic scoliosis (AIS)** study.

Here's how we'll proceed for **each patient case**:

✅ My Role:

Upon receiving **AP, lateral, and right/left bending full-spine radiographs**, I will:

1. 📏 **Measure Cobb angles**:
   - Identify major and minor curves.
   - Measure angles using standard method (end vertebrae → angle between their upper/lower endplates).
2. 🔢 **Perform full Lenke classification**:
   - **Curve type (1–6)**
   - **Lumbar modifier (A, B, C)**
   - **Sagittal modifier (-, N, +)**

     ➤ This requires lateral radiograph (to assess thoracic kyphosis from T5–T12).

Ask anything

ChatGPT can make mistakes. Check important info.

ChatGPT 4o

3. 🧭 **Provide surgical planning suggestion:**
   - Recommend **fusion levels** per Lenke guidelines (e.g., neutral vertebra to neutral vertebra).
   - Consider structurality of curves (from bending films).
   - Highlight selective vs. non-selective fusion decisions if relevant.

📩 What I Need From You:

For each patient, please upload the **3 standard radiographic views** (at minimum):

- **Standing AP**
- **Standing lateral**
- **Right and left side-bending films** (preferably full spine, if available)

Optional but helpful:

- Traction film (if any)
- Clinical notes (age, Risser, etc.)

🔁 Format of My Response:

Each patient will be reported like this:

pgsql

Copy code

```
Patient 1:
1. Cobb angle(s):
   - MT T5–T11: ~54°, right (structural)
   - TL/L T11–L4: ~43°, left (non-structural)
2. Lenke classification: Type 1CN
3. Surgical recommendation: Posterior spinal fusion from T4 to T12 (neutral-stable vertebra)
```

Ask anything

ChatGPT can make mistakes. Check important info.

ChatGPT 4o

✅ Please upload the first case (radiographs) when ready.

📌 I'll maintain a **consistent format** for all cases so you can easily transfer the data into your manuscript or Excel sheet later.

Looking forward to working with you on this!

You will provide the results for each patient in the following format: (1) Cobb angles: first the curve location (PT/MT/TL/L), then the vertebral levels and the magnitude of the curve (for example, 40°), followed in parentheses by whether the curve is structural or nonstructural. Example: PT: T2–T6: 33° (S), MT: T6–T12: 59° (S), L: L1–L4: 39° (NS). (2) Lenke classification: you will report the Lenke classification each time as a single term that includes all three parameters. Example: 2BN. (3) Suggested surgical fusion levels: in line with the Lenke recommendations, you will propose the appropriate fusion levels. Example: T2–L2.

Understood. I will provide the results **exactly** in the format you've specified for each AIS patient. Here's a summary of the standardized format I will use for every case you send:

✅ Report Format Per Patient:

1. Cobb angles:

- Each curve labeled by location:
  - PT (Proximal Thoracic)
  - [illegible]

Ask anything

ChatGPT can make mistakes. Check important info.

ChatGPT 4o

✅ **Report Format Per Patient:**

1. Cobb angles:

- Each curve labeled by location:
  - PT (Proximal Thoracic)
  - MT (Main Thoracic)
  - TL (Thoracolumbar)
  - L (Lumbar)
- Structure:
  - Indicate **structural (S)** or **non-structural (NS)** based on side-bending radiographs.
- Format:

```yaml
PT: T2–T6: 33° (S), MT: T6–T12: 59° (S), L: L1–L4: 39° (NS)
```

2. Lenke classification:

- Must include:
  - Curve type (1–6)
  - Lumbar modifier (A/B/C)
  - Sagittal modifier (-/N/+)
- Format:

```
2BN
```

3. Suggested surgical fusion levels:

- Based on Lenke fusion guidelines (neutral/stable [illegible]tebra principle, selective fusion rules).
- Format:

Copy code

Ask anything

ChatGPT can make mistakes. Check important info.

ChatGPT 4o

3. Suggested surgical fusion levels:

- Based on Lenke fusion guidelines (neutral/stable vertebra principle, selective fusion rules).
- Format:

Copy code

```
T2–L2
```

📩 Please proceed by uploading the first patient's radiographs (AP, lateral, and right/left bending views).

I'm ready to begin.

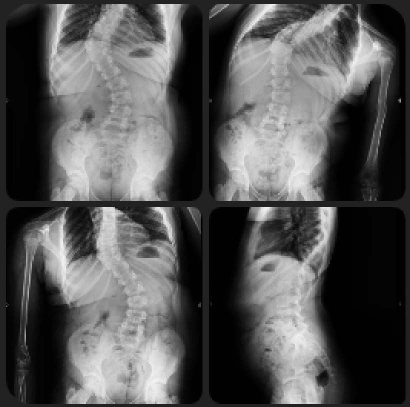

Here is the detailed evaluation for the first AIS patient based on the provided radiographs (standing AP, lateral, and side-bending views):

Ask anything

ChatGPT can make mistakes. Check important info.

ChatGPT 4o

✅ **Patient 1:**

1. Cobb angles:

- **MT: T5–T11: 52° (S)**
- **TL/L: T11–L4: 34° (S)**

📝 *Bending films confirm both curves are structural (remain >25°). The thoracolumbar/lumbar curve does not correct sufficiently on side bending.*

---

2. Lenke classification:
3CN

- **Type 3:** Double major (MT and TL/L both structural)
- **Lumbar modifier C:** Central sacral vertical line (CSVL) falls entirely to the concave side of the lumbar curve
- **Sagittal modifier N:** Normal thoracic kyphosis (~20° on lateral T5–T12)

---

3. Suggested surgical fusion levels:
T4–L4

- Upper instrumented vertebra (UIV): T4 (to include PT region, proximal to upper-end of MT curve)
- Lower instrumented vertebra (LIV): L4 (to encompass structural lumbar curve based on neutral and stable vertebra principles)

📗 If you'd like, I can continue compiling these in Excel or a structured table.
📩 Please upload the next patient's images when rea[illegible]

Ask anything

ChatGPT can make mistakes. Check important info.

Hello. I am conducting a study on scoliosis measurement, classification, and surgical planning in adolescent idiopathic scoliosis patients. We will perform measurements together in anonymous adolescent idiopathic scoliosis patients. I will send you the scoliosis AP, lateral, and right/left bending plain radiographs of anonymous patients, and you will (1) measure the scoliosis Cobb angles, (2) determine the Lenke classification, and (3) based on the Lenke recommendations, provide a suggestion for the surgical levels if surgery were to be planned for these patients.

Claude 3.7 Sonnet

Hello! I'd be happy to help with your study on adolescent idiopathic scoliosis (AIS). Your plan to analyze radiographs for Cobb angle measurements, Lenke classification, and surgical level recommendations is a comprehensive approach to scoliosis assessment.

For each patient case, I can:

1. Measure the scoliosis Cobb angles from the provided radiographs
2. Determine the Lenke classification (types 1-6 with lumbar and sagittal modifiers)
3. Provide evidence-based surgical level recommendations following Lenke guidelines

To get started, please upload the AP (anteroposterior), lateral, and right/left bending radiographs for your first patient. I'll use the `data_analysis` tool to process these images and perform the necessary measurements and analyses.

A few quick notes:

- For accurate Cobb angle measurement, I'll identify the most tilted vertebrae at the upper and lower ends of each curve
- Lenke classification requires assessment of curve flexibility from bending films and sagittal alignment from lateral views
- Surgical recommendations will be based on curve magnitude, flexibility, and the specific Lenke curve type

Claude 3.7 Sonnet

Ask anything, @ models, / prompts

Think Tools

Feel free to upload the first set of radiographs whenever you're ready, and I'll begin the analysis.

You will provide the results for each patient in the following format: (1) Cobb angles: first the curve location (PT/MT/TL/L), then the vertebral levels and the magnitude of the curve (for example, 40°), followed in parentheses by whether the curve is structural or nonstructural. Example: PT: T2–T6: 33° (S), MT: T6–T12: 59° (S), L: L1–L4: 39° (NS). (2) Lenke classification: you will report the Lenke classification each time as a single term that includes all three parameters. Example: 2BN. (3) Suggested surgical fusion levels: in line with the Lenke recommendations, you will propose the appropriate fusion levels. Example: T2–L2.

Claude 3.7 Sonnet

I understand the format you'd like me to use for reporting results. For each patient case, I will provide:

1. **Cobb Angles**:
   - Format: Curve location (PT/MT/TL/L) + vertebral levels + magnitude in degrees + structural status (S/NS)
   - Example: PT: T2–T6: 33° (S), MT: T6–T12: 59° (S), L: L1–L4: 39° (NS)
2. **Lenke Classification**:
   - Format: Single term with all three parameters (curve type 1-6, lumbar modifier A/B/C, thoracic sagittal modifier -/N/+)
   - Example: 2BN
3. **Suggested Surgical Fusion Levels**:
   - Format: Starting vertebra–ending vertebra
   - Example: T2–L2

Claude 3.7 Sonnet

Ask anything, @ models, / prompts

Think Tools

Once you upload the radiographs for a patient, I'll analyze them and provide the assessment in this exact format. The Lenke classification system combines curve type (1-6), lumbar spine modifier (A, B, or C), and thoracic sagittal modifier (-, N, or +) to guide treatment decisions.

I'm ready to begin the analysis whenever you upload the first set of radiographs (AP, lateral, and right/left bending views).

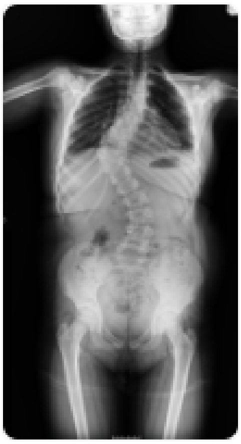

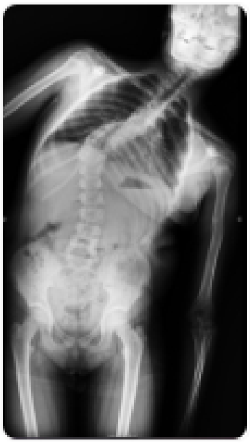

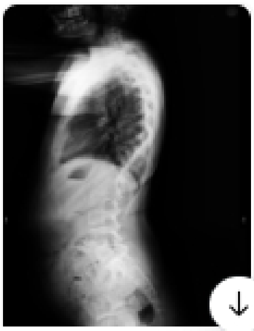

Claude 3.7 Sonnet

Ask anything, @ models, / prompts

Think Tools

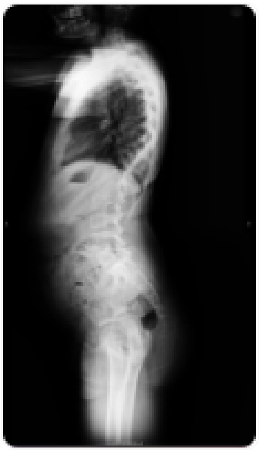

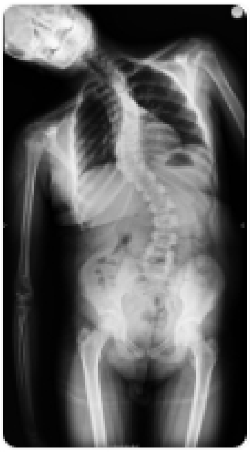

Patient- 1

Claude 3.7 Sonnet

Analysis of Patient-1 Radiographs

Based on the provided radiographs (AP standing, lateral, and bending views), here is my assessment:

1. Cobb Angles:

- MT: T5-T12: 48° (S)
- L: L1-L4: 32° (NS)

Claude 3.7 Sonnet

Ask anything, @ models, / prompts

Think Tools

Claude 3.7 Sonnet

Analysis of Patient-1 Radiographs

Based on the provided radiographs (AP standing, lateral, and bending views), here is my assessment:

1. Cobb Angles:

- MT: T5-T12: 48° (S)
- L: L1-L4: 32° (NS)

2. Lenke Classification: 1CN

3. Suggested Surgical Fusion Levels: T5-L1

Assessment Explanation:

From the radiographs, I can identify a main thoracic curve (MT) which appears structural and is the primary curve. The lumbar curve is compensatory and appears to correct on bending films. The sagittal alignment shows normal kyphosis in the thoracic spine. Based on the Lenke classification system, this represents a Type 1 curve (main thoracic) with a C lumbar modifier (centerline of L4 vertebra falls between pedicles of the lumbar spine) and N sagittal modifier (normal kyphosis).

The fusion levels recommendation follows Lenke guidelines for Type 1 curves, where selective thoracic fusion is appropriate. The upper instrumented vertebra (UIV) is at the upper end vertebra (T5), and the lower instrumented vertebra (LIV) is at the stable vertebra (L1), as this appears to be the first vertebra touched by the center sacral vertical line (CSVL).

Compare with GPT-5.1

Claude 3.7 Sonnet

Ask anything, @ models, / prompts

Think Tools
