# Supplementary material for: Reliability of Large Language Model-Based Artificial Intelligence in AIS Assessment: Lenke Classification and Fusion-Level Suggestion
Source: Diagnostics (Basel). 2025 Dec 16;15(24):3219. doi: 10.3390/diagnostics15243219 (PMC12731402; doi:10.3390/diagnostics15243219)
Supplement: Supplementary file 1 [file diagnostics-15-03219-s001.zip › Supplementary Table S1.pdf]

Supplementary Table S1. Subtype-Specific Accuracy of AI Models in Lenke Classification

**Lenke Subtype**	**ChatGPT Accuracy (%)**	**Claude Accuracy (%)**	**DeepSeek Accuracy (%)**	**Gemini Accuracy (%)**
1AN	18.2	18.2	13.6	0.0
1BN	14.3	42.9	14.3	0.0
1CN	0.0	14.3	0.0	0.0
1C–	0.0	0.0	0.0	0.0
1A+	0.0	0.0	0.0	0.0
2AN	0.0	0.0	0.0	0.0
2BN	0.0	0.0	14.3	0.0
2B+	0.0	0.0	0.0	0.0
2CN	0.0	0.0	0.0	0.0
2C+	0.0	0.0	0.0	0.0
3AN	0.0	0.0	0.0	0.0
3BN	0.0	0.0	0.0	0.0
3B+	0.0	0.0	0.0	0.0
3C+	0.0	0.0	0.0	0.0
4BN	0.0	0.0	0.0	0.0
4C+	0.0	0.0	0.0	0.0
5AN	0.0	0.0	0.0	0.0
5BN	0.0	0.0	0.0	0.0
5C+	0.0	0.0	0.0	0.0
5CN	0.0	0.0	0.0	0.0
6AN	0.0	0.0	0.0	0.0
6BN	0.0	0.0	0.0	0.0
6C+	0.0	0.0	0.0	0.0
6CN	0.0	0.0	0.0	0.0

Footnote:

Accuracy = proportion of exact matches between AI output and surgeon-derived subtype. Partial correctness is not clinically meaningful in the Lenke system.
