# Supplementary figures and images for: Reliability of Large Language Model-Based Artificial Intelligence in AIS Assessment: Lenke Classification and Fusion-Level Suggestion
Source: Diagnostics (Basel). 2025 Dec 16;15(24):3219. doi: 10.3390/diagnostics15243219 (PMC12731402; doi:10.3390/diagnostics15243219)

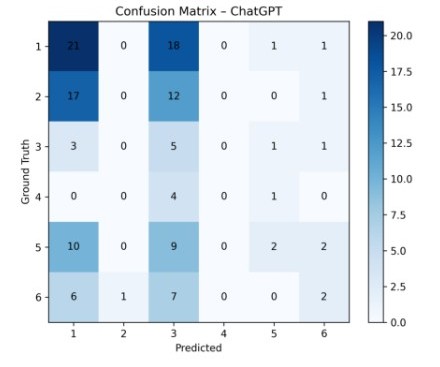

Supplement: Supplementary file 1 [file diagnostics-15-03219-s001.zip › Supplementary Figure S1.jpg]

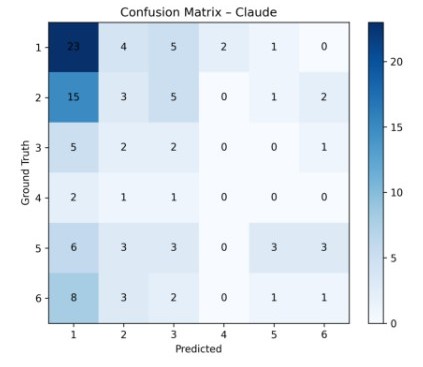

Supplement: Supplementary file 1 [file diagnostics-15-03219-s001.zip › Supplementary Figure S2.jpg]

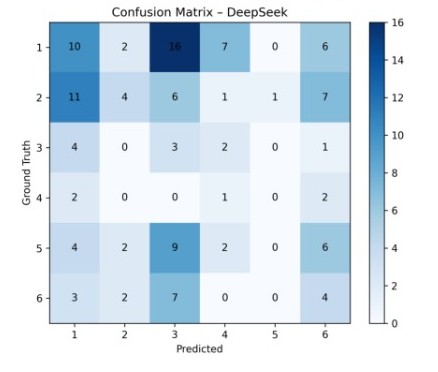

Supplement: Supplementary file 1 [file diagnostics-15-03219-s001.zip › Supplementary Figure S3.jpg]

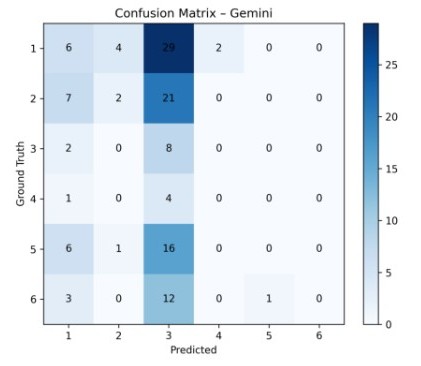

Supplement: Supplementary file 1 [file diagnostics-15-03219-s001.zip › Supplementary Figure S4.jpg]
